# Neuroprotective effects of *Eucommia ulmoides* Oliv. and its bioactive constituent work via ameliorating the ubiquitin-proteasome system

**DOI:** 10.1186/s12906-015-0675-7

**Published:** 2015-05-21

**Authors:** Hong Guo, Fang Shi, Meijiao Li, Qingqing Liu, Bin Yu, Limin Hu

**Affiliations:** Institute of Traditional Chinese Medicine, Tianjin University of Traditional Chinese Medicine, #312 Anshan Xi Road, Nankai District, Tianjin, 300193 China

**Keywords:** *Eucommia ulmoides*, Parkinson’s disease, Ubiquitin-proteasome system

## Abstract

**Background:**

Parkinson’s disease (PD) is a chronic neurodegenerative disorder characterized by a loss of dopaminergic neurons in the substantia nigra, decreased striatal dopamine levels, and consequent extrapyramidal motor dysfunction. The purpose of this study was to investigate potential in vivo protective effects of Duzhong against 1-methyl-4-phenyl-1,2,3,6-tetrahydropyridine (MPTP), as well as the bioactive constituents against 1-methyl-4-phenylpyridinium (MPP^+^) toxicity in vitro.

**Methods:**

Male C57BL/6 mice were intraperitoneally administrated five consecutive injections of MPTP every 24 h at a dose of 30 mg/kg to induce an in vivo PD model. Pole and traction tests were performed in mice to evaluate motor deficits and bradykinesia after the final MPTP administration. The striatal levels of dopamine and its metabolites, 3,4-dihydroxyphenylacetic acid and homovanilic acid, were measured using a High-performance liquid chromatography-electrical conductivity detector. To further explore the bioactive constituents and protective mechanisms of Duzhong, seven compounds from Duzhong were tested on MPP^+^-treated SH-SY5Y cell lines in vitro. A proteasome enzymatic assay and Cell Counting Kit-8 were performed to examine proteasomal activity and cell viability of Duzhong-treated cells, respectively, after exposure to MPP^+^ and proteasome inhibitor MG132.

**Results:**

Duzhong antagonized the loss of striatal neurotransmitters and relieved the associated anomaly in ambulatory locomotor activity in PD mice after a 3-day pre-treatment of Duzhong crude extract. The five Duzhong compounds attenuated MPP^+^-induced dysfunction of protease activity and reduced MG132-induced cytotoxicity.

**Conclusion:**

Duzhong could serve as a potential candidate for PD treatment, and its mechanism involves the amelioration of the ubiquitin-proteasome system.

## Background

Parkinson’s disease (PD) is the second most common neurodegenerative disease after Alzheimer’s disease. It affects people aged over 55 years with psychological and physical manifestations [1, 2]. Pathologically, it is marked by the extensive loss of dopaminergic neurons in the substantia nigra, which result in extrapyramidal motor dysfunction, including tremor, rigidity, and bradykinesia [3]. Although the etiology of PD is incompletely understood, increasing evidence indicates that oxidative stress, mitochondrial dysfunction, and ubiquitin-proteasome system (UPS) dysfunction may play a role in the principal molecular pathways of PD [4, 5].

Impairment of the UPS is a cellular mechanism underlying the neurodegenerative process of PD. Pathologically, it is characterized by the substitution of a highly soluble native neuronal protein with a progressively polymerized protein, which forms to an altered conformation. This results in intracellular aggregation, a process associated with neuronal dysfunction and loss. The abnormal or misfolded proteins are normally targeted via ubiquitination to the proteasome, where they are degraded in an adenosine triphosphate-dependent manner [6]. Therefore, the function of a protein degradation system, especially UPS, is a promising target in studying the mechanisms of PD and the development of potential therapeutic drugs. Two gene mutations, which are unstable in the UPS and contribute to the demise of dopaminergic neurons, encode parkin [7] and ubiquitin C-terminal hydrolase-L1 (UCH-L1) [8]. The parkin gene contains an N-terminal ubiquitin-like domain and a C-terminal RING domain comprising two RING finger motifs [5]. Like many other RING finger-containing proteins, parkin can function as an E3 ubiquitin protein ligase [9], which is an important part of the cellular machinery that covalently tags target proteins with ubiquitin [10]. UCH-L1 belongs to a family of deubiquitinating enzymes, which might function as a dimerization-dependent ubiquitin protein ligase and can maintain ubiquitin homeostasis by promoting stability of ubiquitin monomers [11, 12].

One way to study idiopathic PD in animals models involves the use of 1-methyl-4-phenyl-1,2,3,6-tetrahydropyridine (MPTP), a precursor of the mitochondrial toxin 1-methyl-4-phenylpyridinium (MPP^+^), which targets nigrostriatal dopaminergic neurons in the substantia nigra in a pattern precipitating pathogenesis analogous to human PD [13]. MPP^+^ mediates dopaminergic degeneration by inhibiting electron transport chain activity at complex I [14]. Loss of oxidative metabolism and deficient production of adenosine-triphosphate through the electron transport chain can bring about rapid neuronal depolarization and a calcium-mediated cascade of cell death [15, 16].

Herbal medicines are becoming more popular for improving quality of life, with limited or no side effects [17]. Currently, levodopa has been considered to be the most clinically efficacious drug for PD treatment. However, long-term treatment with levodopa leads to dyskinesia [18, 19]. Therefore, alternative treatments for PD, such as traditional Chinese medicine, were investigated. Duzhong is from the bark of *Eucommia ulmoides* Oliv., and it has been widely used in traditional Chinese medicine to treat hypertension [20, 21]. Modern molecular research has shown that Duzhong prevents/decreases hydrogen peroxide and amyloid β-induced neuronal cytotoxicity [22, 23] as well as improves learning and memory [24]. It also protects neuronal cells from apoptosis induced by the PD-related neurotoxin 6-hydroxydopamine in SH-SY5Y cells [25], and improves scopolamine-induced learning and memory deficits in mice [26]. However, studies evaluating the neuroprotective role of Duzhong in MPP^+^/MPTP PD experimental models have not been conducted. In the present study, we investigated the potential protective effects of Duzhong in vivo against MPTP, and its bioactive constituents against MPP^+^ toxicity in vitro.

## Methods

### Animals

Specific pathogen-free, adult, male, C57BL/6 mice (25 ± 2 g body weight) were housed in standard cages with wood shavings. Ten animals per cage were kept at room temperature (25 °C) and the light schedule was set for 12 h of light from 8:00 AM to 8:00 PM. Food and water were freely available. The study was conducted with the consent of the Ethics Committee for the use of experimental animals as authorized by the Tianjin University of Traditional Chinese Medicine (Approval number: TCM-LAEC2014007).

### Drugs

The bark of *Eucommia ulmoides* Oliv*.* was purchased from Henan Drug Company (Henan, PR China) and authenticated by Professor Li Tianxiang (Tianjin University of Traditional Chinese Medicine, Tianjin). A voucher specimen (EUL090617) was deposited in the Institute of Traditional Chinese Medicine, Tianjin University of Traditional Chinese Medicine. The dried bark of *E. ulmoides* was refluxed with 95 % ethanol twice (2 h each time), and the ethanol extract was pooled, concentrated, and dried using a vacuum (crude extract, yield 11 %).

**Fig. 1:**
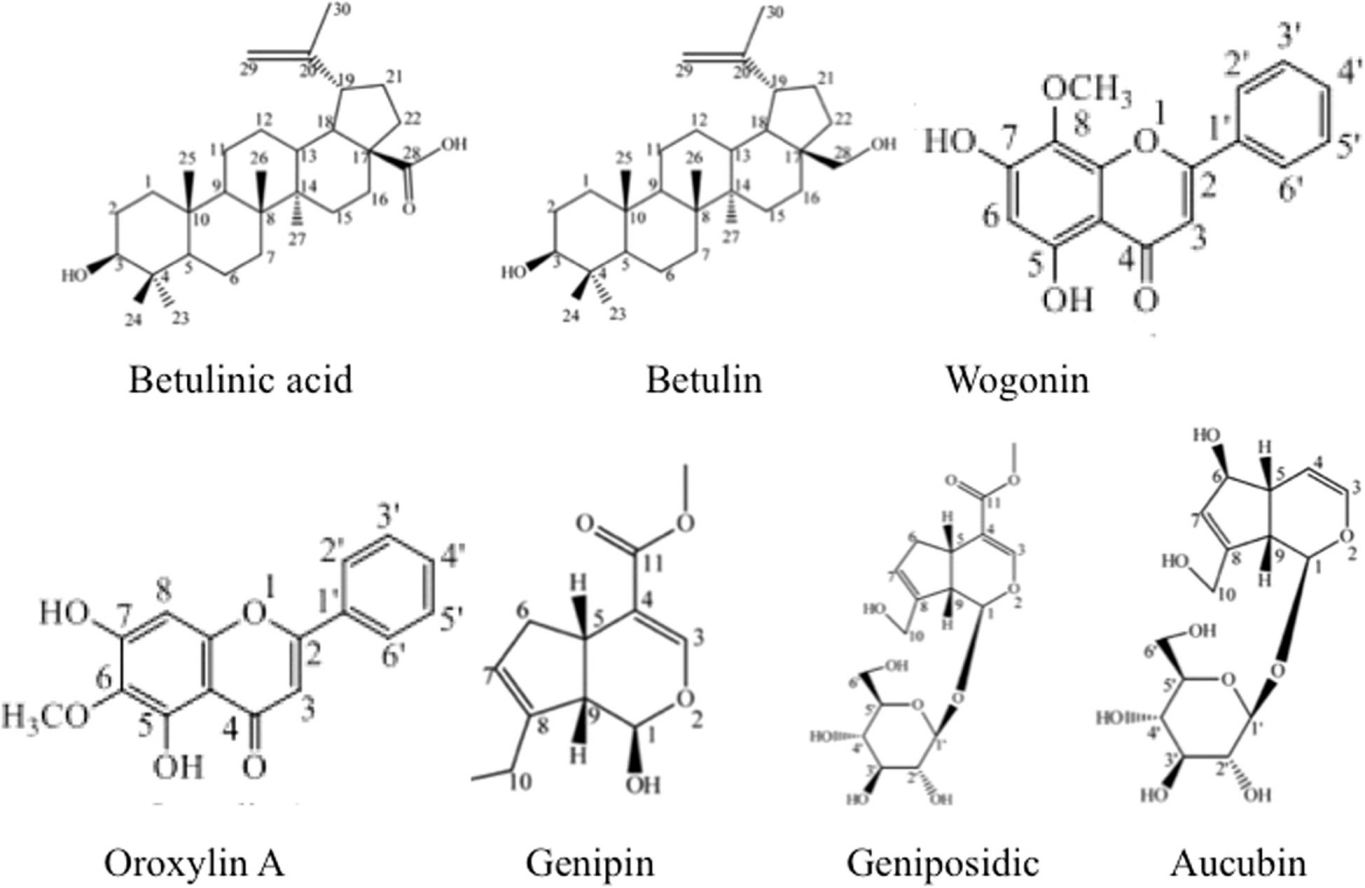
Structures of the seven compounds isolated from *Eucommia ulmoides*. Two terpenes (betulinic acid and betulin), two flavonoids (wogonin and oroxylin A), and three iridoids (genipin, geniposidic, and aucubin) were isolated from *E. ulmoides* and purities (98 %) were verified using HPLC-UV analysis

Professor Su’s group (Tianjin University, Tianjin) isolated 18 compounds from *E. ulmoides* ethanol extracts and identified 16 structures. A detailed isolation procedure of these compounds has been referenced by Yin et al. [27]. We selected seven compounds (Table 1) with molecular weight under 500 g/mol in vitro: betulinic acid (Bea), betulin (Bet), wogonin (Wog), oroxylin A (Oro), genipin (Gen), geniposidic (Ged), and aucubin (Auc). The chemical structures are shown in Fig. 1 and the 98 % purities were verified using High-performance liquid chromatography-ultraviolet detector (HPLC-UV) analysis. All compounds were tested at 10 μM, dissolved in dimethyl sulfoxide at 10 mM as stock solution, and diluted in culture medium.

**Table 1:** Compounds

Compound	Abbreviation	Formula	Molecular weight
Betulinic acid	Bea	C_30_H_48_O_3_	456.71
Betulin	Bet	C_30_H_50_O_2_	442.71
Wogonin	Wog	C_16_H_12_O_5_	284.27
Oroxylin A	Oro	C_16_H_12_O_5_	284.26
Genipin	Gen	C_11_H_14_O_5_	226.23
Geniposidic	Ged	C_17_H_24_O_10_	378.37
Aucubin	Auc	C_15_H_22_O_9_	346.33

### Reagents

Fetal bovine serum (FBS), penicillin, and streptomycin were purchased from Gibco Invitrogen. 1-methyl-4-phenyl-1,2,3,6-tetrahydropyridine (MPTP), 1-methyl-4-phenylpyridinium (MPP^+^) were purchased from Sigma-Aldrich. 1640 medium was purchased from Hyclone. Cell Counting Kit-8 (CCK-8) was purchased from Dojindo. Fluorogenic Suc-LLVY-AMC was purchased from Boston Biochem. All other chemicals were of analytical grade and obtained from standard commercial suppliers. Madopar was purchased from Roche, and each tablet contained 200 mg levodopa and 50 mg benserazide.

### Animal grouping and treatment

Male mice (*n* = 48) were assigned to six groups: control group, MPTP group (MPTP-treatment group), 150 mg/kg group (150 mg Duzhong/kg body weight plus MPTP), 300 mg/kg group (300 mg Duzhong/kg body weight plus MPTP), 600 mg/kg group (600 mg Duzhong/kg body weight plus MPTP), and Madopar group (50 mg Madopar/kg body weight plus MPTP). Duzhong and Madopars were dissolved in 0.5 % sodium carboxymethyl cellulose. DuZhong and Madopar were orally administered at the indicated concentrations every 24 h for 8 consecutive days. To evaluate the effects of Duzhong and Madopar in the PD mouse model, MPTP (30 mg/kg) was intraperitoneally injected with five consecutive doses every 24 h from day 4 to day 8; 1 h later, the mice were orally administered Duzhong and Madopar. An equal volume of saline, instead of MPTP, was injected into the mice in the control group.

### Neurobehavioral assessment

All animals in these studies underwent behavioral analysis after the final MPTP administration. The observer was blinded to all groups until the completion of all behavioral studies.

### Pole test

The pole test for bradykinesia was conducted according to a previously published method, with slight modifications [28]. The mouse was positioned downward at the top of a vertical rough-surfaced pole (1-cm diameter, 80-cm height), and the duration from the time point that the mouse started to move until the time point that the mouse arrived at the floor (total locomotor activity) was recorded. The pole test was performed three times consecutively for each mouse, and the average value was recorded.

### Traction test

The traction test evaluates muscle strength and equilibrium. When the mouse forepaws were placed on a rope, hind limb placements were scored from 1 to 3, with the lowest score indicating the most severe deficit. In short, if both hind limbs seized the rope, the score was 3; if one or no hind limb seized the rope, the score was 2 or 1, respectively.

### Measurement of dopamine, 3,4-dihydroxyphenylacetic acid (DOPAC), and homovanilic acid (HVA) levels in the striatum

After completion of behavioral studies, all animals were sacrificed for striatal catecholamine determination. Dopamine, DOPAC, and HVA were measured as previously described [29]. Briefly, brains were quickly removed, the left and right striatum were quickly dissected from the brain, weighed, and sonicated in chilled 0.1 mol/L perchloric acid (per 1 mg tissue plus 10 mL perchloric acid). The suspension was then vortexed, and the striatum was centrifuged at 4 °C at 14,000 r/min for 20 min. The clear supernatant was collected and stored at −80 °C until further HPLC analysis. Ten microliters of supernatant was injected directly into the HPLC-ECD system using a Waters C18 reversed-phase analytic column. The mobile phase contained 3 % acetonitrile, 19 % methanol, and 78 % water (11.56 mmol/L octane sulfonic acid sodium, 100 mmol/L NaH_2_PO_4_, 0.095 mmol/L EDTA-2Na).

### Cell culture

Human neuroblastoma cells (SH-SY5Y) were purchased from the Cell Center of the Institute of Basic Medical Science Research (Chinese Academy of Medical Sciences, Beijing, China). SH-SY5Y cells were cultured in 1640 supplemented with 15 % FBS, 100 U/mL penicillin, and 100 μg/mL streptomycin. The medium was replaced every 2 days. Cells were maintained at 37 °C and 5 % CO_2_.

### Proteasomal peptidase activity assay

The proteasome enzymatic assay was performed as previously described [30]. Briefly, SH-SY5Y cells were split into 48-well plates at a density of 1 × 10^5^/mL, and exposed to 1 mM MPP^+^ with 10 μM of each compound for 24 h. After treatment, yhr cells were collected, washed, and lysed with hypotonic buffer (10 mM HEPES, 5 mM MgCl_2_, 10 mM KCl, 1 % sucrose, snf 0.1 % CHAPS). Lysates were then incubated with fluorogenic Suc-LLVY-AMC (75 μM) in assay buffer (50 mM Tris–HCl, 20 mM KCl, 5 mM MgOAc, and 10 mM DTT, pH 7.6) at 37 °C or 30 min. The cleaved fluorescent product was measured at the excitation wavelength of 380 nm and emission wavelength of 460 nm using a fluorescent microplate reader (Flex Station 3, Molecular Devices, CA, USA).

### Viability assay

Cell viability was evaluated using a CCK-8 assay by measuring absorbance. Cells were incubated with 200 μM MG132, a potent reversible inhibitor of the proteasome, together with 10 μM of each compound for 24 h. Then, culture medium was removed and 10 % CCK-8 was added to each well. Cells were then incubated for 30 min at 37 °C, and the absorbance was measured at 450 nm using a microplate reader (Spectra III, Tecan, Switzerland).

### Statistical analysis

Data were expressed as mean ± SD. Statistical analysis was performed using one-way analysis of variance, as well as the least significant difference test, using SPSS 13.0 software. *P* < 0.05 was considered statistically significant.

## Results

### Effect of Duzhong on neurobehavior in MPTP-treated mice

**Fig. 2:**
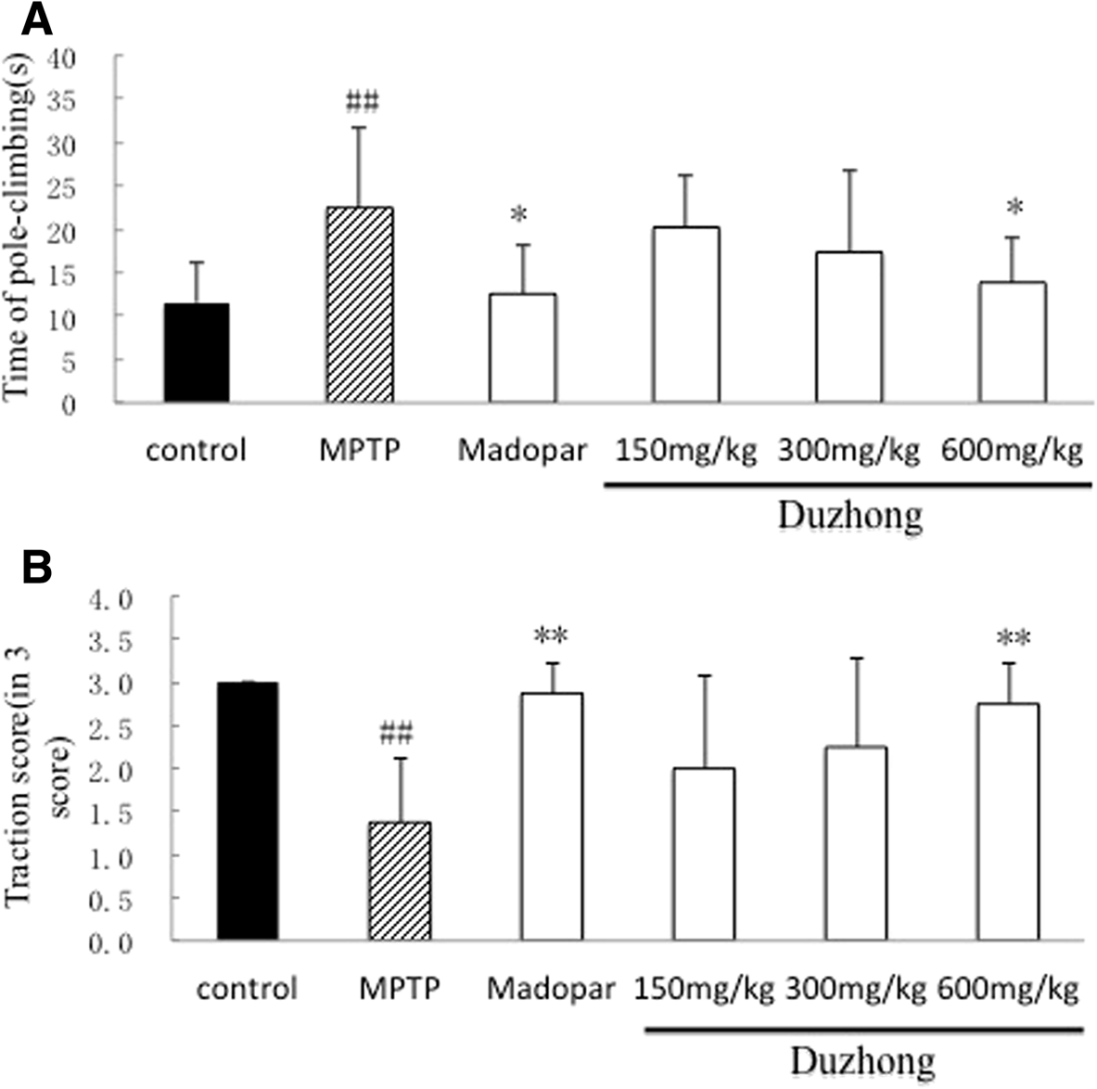
Protective effect of Duzhong against MPTP-induced behavioral dysfunction on a mouse model of Parkinson’s disease. The time period from the final MPTP injection until the animal arrived on the floor (total locomotor activity) (**a**) was recorded with a limit of 30 s for the pole test. The traction test measured hind limb grip power (**b**). Total locomotor activity time was significantly prolonged, and strength on the traction test was decreased in MPTP-treated mice. Madopar and 600 mg/kg Duzhong significantly shortened the time to reach the platform and improved the hind limb grip score. ^##^ *p* < 0.01 vs. control group, *p < 0.05, **p < 0.01 vs. MPTP group, *n* = 8 animals/group

We performed the pole and traction tests to evaluate motor deficits and bradykinesia, respectively, in MPTP-treated mice. The pole test showed that total locomotor activity time was significantly prolonged after subchronic MPTP treatment. Prophylactic treatment with Duzhong (150, 300, and 600 mg/kg) shortened the time needed to reach the platform, and results showed that 600 mg/kg was the most effective (Fig. 2a). These results suggested that Duzhong prevented MPTP-induced bradykinesia. MPTP-treated mice showed decreased strength on the traction test, but prophylactic treatment with Duzhong (150, 300, and 600 mg/kg) resulted in improved traction test performance, suggesting that the initial lesions caused by MPTP were prevented by prophylactic treatment with Duzhong (Fig. 2b).

### Duzhong blocks MPTP-induced loss of striatal dopamine and its metabolites

**Fig. 3:**
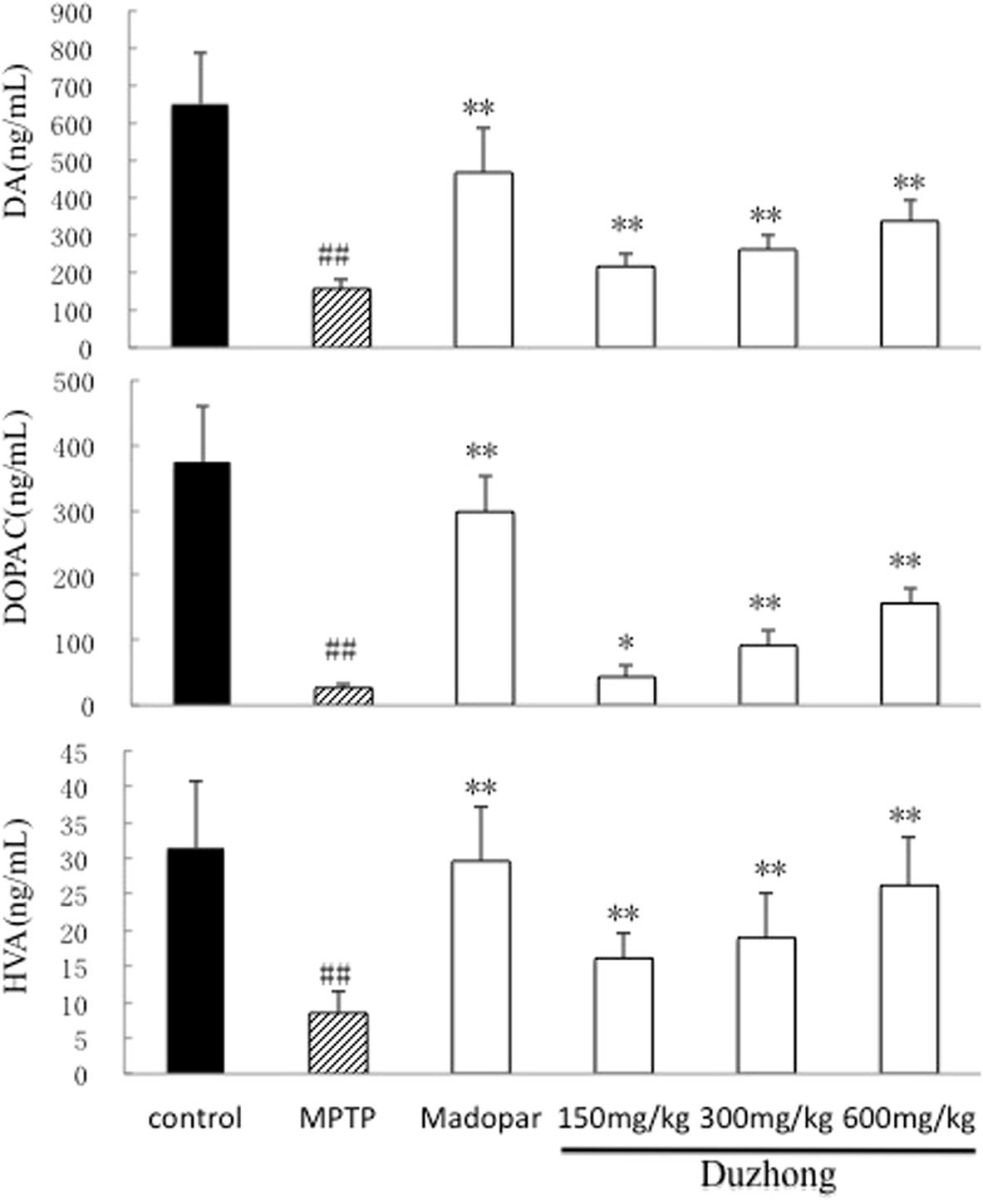
Effects of Duzhong on striatum dopamine, DOPAC, and HVA levels in MPTP-treated mice. Striatum dopamine, DOPAC, and HVA levels were measured by HPLC-ECD. MPTP treatment significantly reduced striatal dopamine, DOPAC, and HVA levels. Duzhong treatment blocked the MPTP-induced decrease of striatal dopamine and its metabolites in a dose-dependent manner. ^##^ *p* < 0.01 vs. control group, *p < 0.05, **p < 0.01 vs. MPTP group, n = 8 animals/group

We measured striatal levels of dopamine and its metabolites, DOPAC and HVA, by HPLC-ECD. MPTP treatment significantly reduced striatal dopamine, DOPAC, and HVA levels. Duzhong (150, 300, and 600 mg/kg) treatment blocked the MPTP-induced decrease of striatal dopamine and its metabolites in a dose-dependent manner (Fig. 3). These results indicated that Duzhong pretreatment improved dopamine, DOPAC, and HVA levels in the striatum of experimental PD mice.

### Up-regulation of proteasomal activity by Duzhong compounds

**Fig. 4:**
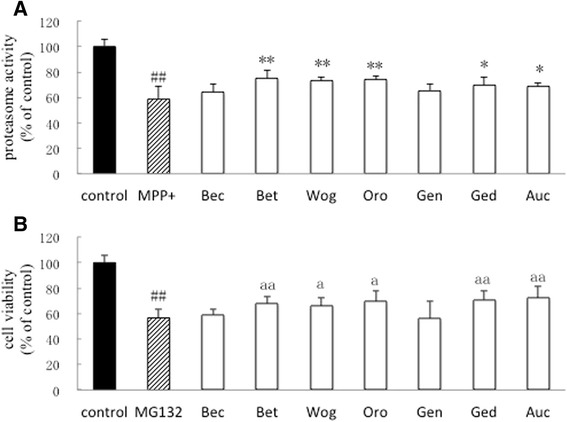
Effect of Duzhong compounds on MPP^+^ or MG132-induced neurotoxicity in the dopaminergic neuroblastoma SH-SY5Y cell line. Cells were exposed to 10 μM compound and 1 mM MPP^+^ or 200 μM MG132 for 24 h. Proteasomal peptidase activity (**a**) and cell viability (**b**) of SH-SY5Y cells were examined. Following treatment with seven compounds of Duzhong (10 μM each), five of the compounds (Bet, Wog, Oro, Ged, and Auc) significantly reversed MPP^+^-induced reduction in proteasome activity and attenuated the MG132-induced dysfunction of protease activity and cytotoxicity. Data are percentages of the control group. ^##^ *p* < 0.01 vs. control group, *p < 0.05, **p < 0.01 vs. MPP^+^ group, ^a^ *p* < 0.05, ^aa^ *p* < 0.01 vs. MG132 group, *n* = 6

To determine the proteasomal activity in dopaminergic-characteristic SH-SY5Y cells, a proteasome enzymatic assay was used. Fig. 4a shows that MPP^+^ decreased proteasome activity after 24 h treatment compared with the control group. Treatment with Duzhong compounds (Bet, Wog, Oro, Ged, and Auc) significantly reversed the MPP^+^-induced reduction in proteasome activity.

### Effect of Duzhong compounds on cell death induced by proteasome inhibitors

We further examined whether inhibition of the UPS enhanced MG132 toxicity. We added 200 μM MG132, a potent reversible proteasome inhibitor, to SH-SY5Y cells pretreated with Duzhong compounds for 24 h. Cell survival, as indicated by the CCK-8 method, markedly decreased in the presence of MG132 as shown in Fig. 4b. Five out of seven compounds from Duzhong (Bet, Wog, Oro, Ged, and Auc) attenuated the MG132-induced dysfunction of protease activity and cytotoxicity at a concentration of 10 μM. These results suggested that impairment of the UPS by MG132 was attenuated by Duzhong in SH-SY5Y cells.

## Discussion

In the present study, we tested whether *E. ulmoides* extracts protected against motor function reductions in MPTP-induced mice. Behavioral analysis was conducted after the final injection. The results from the pole test and traction test revealed that Duzhong (150, 300, and 600 mg/kg) increased motor activity of MPTP-treated mice, and 600 mg/kg was the most effective (Fig. 2a).

Neurotransmitter secretion maintains normal neural functions, although neuronal damage can affect neurotransmitter release [31]. Dopamine and its metabolites are the primary neurotransmitters involved in motor functions. Loss of these neurotransmitters directly affects physical movements and is considered a signature feature of PD in humans or in animal models of the disease [32]. In this study, the HPLC-ECD method was used to detect the effects of Duzhong on striatal dopamine, DOPAC, and HVA in PD mice. Treatment with Duzhong (150, 300, and 600 mg/kg) blocked the MPTP-induced decrease of striatal dopamine and its metabolites in a dose-dependent manner. The above findings suggest that Duzhong has beneficial effects on the PD mice model. However, its mechanisms and bioactive constituents have not yet been characterized.

The UPS is essential to all eukaryotic cells [33], and impairment elucidates mutations of key genes, including α-synuclein, Parkin, and UCH-L1, some of which are important in protein processing and degradation [34]. To date, a close relation of PD with a dysfunctional UPS has been demonstrated.

Su et al. isolated 18 compounds from ethanol extracts of *E. ulmoides* and identified structures of 16 compounds, including two lignans, four iridoids, three flavonoids, two triterpenoids, four anthraquinones, and a sterol [35]. The present study selected seven compounds with a molecular weight under 500 g/mol to investigate the protective mechanisms: betulinic acid (Bea), betulin (Bet), wogonin (Wog), oroxylin A (Oro), genipin (Gen), geniposidic (Ged), and aucubin (Auc). The chemical structures of these compounds are shown in Fig. 1. All tested dosages of these compounds were at 10 μM. Results demonstrated that some of the compounds isolated from Duzhong, Bet, Wog, Oro, Ged, and Auc, increased proteasome activity in MPP^+^-treated SH-SY5Y cells, suggesting that toxic accumulation of intracellular proteins was detrimental to neuronal cells and aberrant protein homeostasis was prevented by Duzhong. In addition, MG132, a potent reversible inhibitor of the proteasome, specifically inhibits proteasomal peptidase activity and leads to cell damage. This inhibition was remarkably attenuated by 10 μM Bet, Wog, Oro, Ged, or Auc. These data indicate that Duzhong was involved in the amelioration of the UPS-dependent proteolysis system. The bioactive constituents of Duzhong that were involved in improving proteasome activity included Bet, Wog, Oro, Ged, and Auc. In addition, Duzhong may impact oxidative stress, which is also a major contributor to PD pathology. However, further studies are needed to elucidate the involved mechanisms.

Our study tested seven compounds, including two terpenes (Bea and Bet), two flavonoids (Wog and Oro), and three iridoids (Gen, Ged, and Auc). Among them, a terpene, two flavonoids, and two iridoids showed reduced dysfunction of protease activity. Considering that the UPS involves many different proteins, including ubiquitin, ubiquitin-activating enzyme (E1), ubiquitin-conjugating enzyme (E2), ubiquitin-protein ligase (E3), and its substrate, it is possible that different compounds target different proteins. Duzhong extract is a multi-component mixture. Therefore, Duzhong might act on multiple targets, thereby causing synergistic effects in vivo. To test this hypothesis, further studies are needed.

## Conclusions

These results suggest that Duzhong might serve as an alternative and complementary treatment for PD and its action mechanisms might be mediated by ameliorating the UPS.
